# Biological effectiveness of very high gamma dose rate and its implication for radiological protection

**DOI:** 10.1007/s00411-020-00852-z

**Published:** 2020-06-01

**Authors:** Dante Olofsson, Lei Cheng, Rubén Barrios Fernández, Magdalena Płódowska, Milagrosa López Riego, Pamela Akuwudike, Halina Lisowska, Lovisa Lundholm, Andrzej Wojcik

**Affiliations:** 1 Department of Molecular Biosciences, Centre for Radiation Protection Research, The Wenner-Gren Institute, Stockholm University, Svante Arrhenius väg 20C, 106 91 Stockholm, Sweden; 2 Department of Radiobiology and Immunology, Institute of Biology, Jan Kochanowski University, Kielce, Poland

**Keywords:** Dose rate, Gene expression, Micronuclei, DNA damage response

## Abstract

Many experimental studies are carried out to compare biological effectiveness of high dose rate (HDR) with that of low dose rate (LDR). The rational for this is the uncertainty regarding the value of the dose rate effectiveness factor (DREF) used in radiological protection. While a LDR is defined as 0.1 mGy/min or lower, anything above that is seen as HDR. In cell and animal experiments, a dose rate around 1 Gy/min is usually used as representative for HDR. However, atomic bomb survivors, the reference cohort for radiological protection, were exposed to tens of Gy/min. The important question is whether gamma radiation delivered at very high dose rate (VHDR—several Gy/min) is more effective in inducing DNA damage than that delivered at HDR. The aim of this investigation was to compare the biological effectiveness of gamma radiation delivered at VHDR (8.25 Gy/min) with that of HDR (0.38 Gy/min or 0.79 Gy/min). Experiments were carried out with human peripheral mononuclear cells (PBMC) and the human osteosarcoma cell line U2OS. Endpoints related to DNA damage response were analysed. The results show that in PBMC, VHDR is more effective than HDR in inducing gene expression and micronuclei. In U2OS cells, the repair of 53BP1 foci was delayed after VHDR indicating a higher level of damage complexity, but no VHDR effect was observed at the level of micronuclei and clonogenic cell survival. We suggest that the DREF value may be underestimated when the biological effectiveness of HDR and LDR is compared.

## Introduction

In the vast majority of countries worldwide, national systems of radiological protection are based on recommendations of the International Commission on Radiological Protection (ICRP) (https://www.icrp.org). The ICRP bases its recommendations on scientific results that are regularly summarised and reviewed by the United Nations Scientific Committee on the Effects of Atomic Radiation (UNSCEAR) (https://www.unscear.org). The most relevant scientific results come from epidemiological studies that aim at establishing risk estimates for radiation-induced human health effects. Among those, the Life Span Study (LSS) on the atomic bomb survivors from Hiroshima and Nagasaki is particularly important because of its cohort size, broad dose range and excellent dosimetry (Ozasa et al. 2019). Indeed, ICRP´s estimates regarding the risk of radiation-induced cancers are to a large extend based on the LSS (ICRP 103 2007).

The ICRP relies on the LSS results not only when recommending dose limits but also when determining risk factors that are applied to predict potential health effects from such exposure situations as the Chernobyl accident (WHO 2006) and the Fukushima Daiichi accident (WHO 2013). A problem here is the difference in the dose rate and dose: while the atomic bomb survivors received radiation doses instantaneously, people living in areas contaminated by radiation released from the Chernobyl and Fukushima Daiichi nuclear power plants will slowly accumulate doses during their life time. Moreover, while doses from environmental exposures are generally low, risk factors derived from the LSS cohort are strongly influenced by results from the high dose range, where the relationship between the dose and effect is significant. Can risk factors derived from an acute exposure to high doses be used to predict health effects from chronic exposure to low doses? The ICRP assumes that this is not the case and recommends applying a dose and dose rate effectiveness factor (DDREF) of 2 for predicting cancer risks from low and chronic radiation exposures (ICRP 103 2007). A DDREF of 2 means halving the LSS-derived risk factors for a unit dose. Analogously, the Biological Effects of Ionizing Radiation (BEIR) committee of the USA recommends a DDREF of 1.5 (BEIR ). The validity of a DDREF has been questioned (Jacob et al. 2009) and the ICRP is investigating whether, in the light of new scientific results, its use is still justified (Ruhm et al. 2015, 2016). The criticism of DDREF is in part based on results of cell experiments which show that, per unit dose, the level of radiation-induced DNA mutations and stable-type chromosomal aberrations is the same after exposure to radiation at a high and low dose rate (Manesh et al. 2014 and the papers within).

According to UNSCEAR, a low dose is classified as 0.1 Gy or lower and a low dose rate (LDR) is classified as 0.1 mGy/min averaged over 1 h or lower (UNSCEAR 2012). Any dose or dose rate above these values is regarded as high. In cell experiments that aim at comparing the effectiveness of low and high dose rates, a value of ca 1 Gy/min is generally used as representative for high dose rate. Although nowhere clearly stated, this value is based on the most common output of available radiation exposure facilities and also on the fact that this dose rate is used in external beam radiotherapy (Durante et al. 2018; Ling et al. 2010). The International Atomic Energy Agency recommends the dose rate of > ca 0.3 Gy/min for generating calibration curves to be used in retrospective biological dosimetry for estimating doses received as consequence of accidental radiation exposures (IAEA 2011).

The dose rate at which the Hiroshima and Nagasaki survivors were exposed to largely exceeded the value of 1 Gy/min. The mix of gamma radiation and neutrons can be divided into five dose components: prompt primary gamma radiation (dose rate between 1.2 × 10^4^ and 4.2 × 10^6^ Gy/min), prompt neutron radiation (dose rate between 2.4 × 10^3^ and 1.4 × 10^6^ Gy/min), prompt secondary gamma radiation (dose rate between 10 and 410 Gy/min), delayed gamma radiation (dose rate ca 50 Gy/min), and delayed neutron radiation (dose rate ca 0.1 Gy/min) (Ruhm et al. 2018). The major contributors to the total absorbed dose were the prompt secondary gamma radiation and the delayed gamma radiation component, where the dose rate was in the order of tens of Gy/min.

An interesting and relevant question is whether radiation delivered at a dose rate higher than 1 Gy/min (referred to as very high dose rate) has a higher biological effectiveness than when delivered at a dose rate of 1 Gy/min. If this is the case than experiments aiming at testing the validity of DDREF that were carried out at ca 1 Gy/min underestimate its value.

To test the biological effectiveness of very high dose rate, we have carried out experiments with three ^137^Cs gamma radiation sources which deliver doses at 8.25 Gy/min (representative of very high dose rate—VHDR), 0.79 Gy/min and 0.39 Gy/min, the latter two representative of high dose rate (HDR). Different endpoints were analysed in two different cell types: micronuclei and mRNA levels of three known radiation-responsive genes FDXR, GADD45a and MDM2 were analysed in human peripheral blood lymphocytes and micronuclei, 53BP1 foci and clonogenic cell survival were analysed in the human osteosarcoma cell line U2OS which is stably transfected with a plasmid coding for the 53BP1 protein tagged with GFP (green fluorescence protein) (Bekker-Jensen et al. 2005).

## Materials and methods

### Blood samples: collection and irradiation

Experiments were approved by the regional ethical committee in Stockholm (permit number 2010/27-31/1). Human venous blood was collected from two healthy donors: lymphocytes from a female aged 23 were used for gene expression analyses and lymphocytes from a male aged 57 were used for micronucleus analyses. Blood was collected into heparinized tubes (Li-Heparin LH/9 ml, Sarstedt, Germany), aliquoted into 1 ml Eppendorf tubes and irradiated within 3 h post-collection. Prior to irradiation, whole blood samples were incubated at 37 °C for 20 min in small, styrofoam-coated plastic boxes filled with water and irradiated at 37 °C with 1, 2 or 3 Gy of gamma radiation from one of three ^137^Cs sources available at the Stockholm University: (1) Scanditronix (Uppsala, Sweden) delivering a dose rate of 0.39 Gy/min; (2) Gammacell 40 Exactor (AECL, Canada) delivering a dose rate of 0.79 Gy/min and (3) Gammacell 1000 (AECL, Canada) delivering a dose rate of 8.25 Gy/min. All three sources were calibrated using Fricke dosimetry in collaboration with the Swedish National Metrology Laboratory at the Swedish Radiation Safety Authority.

Once irradiated, the samples and controls were transferred to a corresponding blood culture tube containing culture medium that was warmed to 37 °C as described below, separately for the gene expression and the cytokinesis-block micronucleus assay (CBMN). For each assay, three independent experiments were carried out, meaning that blood was drawn on three occasions within a period of several months.

### Blood samples: gene expression analysis by qPCR in leukocytes

Following irradiation, 0.5 ml of whole blood was added to 4 ml complete medium composed of Roswell Park Memorial Institute (RPMI) 1640 medium (Sigma-Aldrich, St Luis, MO, USA) supplemented with 20% fetal bovine serum (FBS, HyClone, Thermo Fisher Scientific, Waltham, MA, USA), 1% PenStrept (10.000 U penicillin and 10 mg streptomycin/ml, Sigma-Aldrich). The medium contained no phytohemagglutinin (PHA). The samples were then incubated overnight at 37 °C for 24 h. Thereafter, leukocytes were selected for by treatment with red blood cell lysis buffer (Roche, Germany) and samples were processed for qPCR.

RNA was prepared using the E.Z.N.A. Total RNA Kit I (Omega Bio-tek). cDNA was synthesised from 300 ng RNA using the High-Capacity cDNA Reverse Transcription Kit (Thermo Fisher Scientific) with random hexamer primers. Primers, cDNA and 5 × HOT FIREPol^®^ EvaGreen^®^ qPCR Supermix (Solis BioDyne) were mixed and real-time PCR reactions were performed in duplicate on a LightCycler^®^ 480, starting at 95 °C for 15 min, followed by 40 cycles of 95 °C for 15 s, 60 °C for 20 s and 72 °C for 20 s. The 2^−ΔΔCt^ method was used for calculation of relative expression and primer specificity was confirmed using melting curve analysis. Primers used were: FDXR for:TGGATGTGCCAGGCCTCTAC, FDXR rev:TGAGGAAGCTGTCAGTCATGGTT; GADD45a for:ACTGCGTGCTGGTGACGAAT, GADD45a rev: GTTGACTTAAGGCAGGATCCTTCCA; MDM2 for:TATCAGGCAGGGGAGAGTGATACA, MDM2 rev: CCAACATCTGTTGCAATGTGATGGAA. For 18S, sequences are given in (Lundholm et al. 2014).

### Blood samples: cytokinesis-block micronucleus assay in lymphocytes

Following irradiation, 0.5 ml of whole blood was added to 4.5 ml of complete medium composed of Roswell Park Memorial Institute (RPMI) 1640 medium (Sigma-Aldrich, St Luis, MO, USA) supplemented with 20% fetal bovine serum (FBS, HyClone, Thermo Fisher Scientific, Waltham, MA, USA), 1% PenStrept (10.000 U penicillin and 10 mg streptomycin/ml, Sigma-Aldrich) and PHA (M form, Gibco).

Cells were incubated at 37 °C and 5% CO_2_ (Heraeus cell incubator, Germany) in culture tubes with loosened caps (BIO-ONE 10 ml, Greiner, Sollentuna, Sweden) for 72 h. 44 h after the culture start cytochalasin B (C6762, Sigma-Aldrich, Stockholm, Sweden) was added at a final concentration of 5.56 µg/ml. Following 72 h of culture time, the cells were harvested, fixed as described in Kryscio et al. (2001). Briefly, cells were centrifuged and resuspended in 0.14 M KCL. Following a 10 min incubation time at room temperature, they were centrifuged again and fixed in fixative 1 (methanol: NaCl: acetic acid, 4.8: 5.2:1) and subsequently in fixative 2 (methanol: acetic acid, 4.8:1). Following two washing steps in fixative 2, cells were dropped onto clean, dry slides and stained for 10 min with 5% Giemsa (Merck, Stockholm, Sweden) in phosphate-buffered saline (PBS) (Gibco 18912-014, Invitrogen, Täby, Sweden).

Microscopic slides were coded before analysis so that the analyses were carried out in a blinded manner. 3 parallel slides were scored per treatment point and experiment. 1000 binucleated cells (BNC) per slide were analysed for micronuclei by a single scorer. 500 cells per slide were analysed for the replication index.

### U2OS cells: culture and irradiation

Human osteosarcoma cells, U2OS, expressing the GFP (green fluorescent protein) tagged repair protein 53BP1 were constructed as described elsewhere (Bekker-Jensen et al. 2005; Lukas et al. 2011). The cells were cultured in Dulbecco Modified Eagles Medium (Sigma-Aldrich, Stockholm, Sweden, D6046) supplemented with 10% Bovine Calf Serum and 1% Penicillin Streptomycin (Sigma-Aldrich, P4333), in a 5% CO_2_ humidified 37 °C incubator. Cells were cultured in the presence of 400 μg/ml Geneticin-G418 (Sigma-Aldrich, A1720) to positively select cells expressing 53BP1-GFP.

Cells were irradiated at room temperature with 0, 1, 2 and 3 Gy of gamma radiation from one of the two ^137^Cs sources: (1) Scanditronix (Uppsala, Sweden) delivering a dose rate of 0.39 Gy/min; and (2) Gammacell 1000 (AECL, Canada) delivering a dose rate of 8.25 Gy/min. The reason for restricting the analysis to the lowest and highest dose rate was that this part of the study aimed at validating the results obtained with the PBMC. Hence, testing the median dose rate of 0.79 Gy/min was not necessary. More details about the conditions at irradiation are described, separately for each assay, in the sections below.

### U2OS cells: 53BP1 foci

Cells were seeded on 22 × 22 mm glass coverslips at a near-confluent density and placed in 6 cm Petri dishes containing 5 ml of medium. Ca 3 h later, cells were exposed to a single dose of 3 Gy at a dose rate of 0.39 Gy/min and 8.25 Gy/min, as described in the section “U2OS cells: culture and irradiation”.

After irradiation, cells were incubated at 37 °C for between 0 min up to 24 h for analyzing repair kinetics. Control cells were kept at 37 °C for 60 min. After incubation, cells on coverslips were fixed with 3% formaldehyde, 2% sucrose in phosphate-buffered saline for 10 min at room temperature, washed with PBS and placed on cavity slides with wells filled with PBS. Images of individual cells were taken within 2 h from the end of incubation using a fluorescent microscope with 100 × oil immersion lens (Nikon Eclipse E800, Nikon, Tokyo, Japan), a Cool Cube 1 CCD camera (Metasystems, Althusheim, Germany) and the image analysis system ISIS (MetaSystems). Details of image acquisition are described elsewhere (Sollazzo et al. 2017). A modified macro written for ImageJ software (Markova et al. 2007), version 1.43u (https://imagej.en.softonic.com/), was used to calculate the area and number of 53BP1 foci. Foci were categorized as small or large foci based on their areas as described in (Sollazzo et al. 2017). In short, foci with an area between 8 and 39 pixels were classified as small, while foci with an area greater than 40 pixels were classified as large. Between 30 and 50 cells per time point were analysed. 3 independent experiments were performed.

### U2OS cells: clonogenic cell survival

Cells were seed out at various densities (between 100 and 400 cells per dish) on 6-cm-diameter plastic dishes and incubated at 37 °C for 3 h to allow attachment. Thereafter, cells were irradiated at room temperature with 0, 0.25, 0.5, 0.75, 1.0, 2.0, 3.0 and 5.0 Gy at a dose rate of 0.39 Gy/min or 8.25 Gy/min, as described in the section “U2OS cells: culture and irradiation”. After irradiation, cells were incubated at 37 °C for 10 days, fixed with methanol and stained for 5 min with a 5% Giemsa solution. The number of colonies per cells was scored using the countPHICS software (Brzozowska et al. 2019). 3 independent experiments were performed.

### U2OS cells: cytokinesis-block micronucleus assay

Cells were seed out at medium density on 6-cm-diameter plastic dishes containing 5 ml of medium to allow exponential growth. 24 h later dishes were irradiated at room temperature with 0, 1, 2 and 3 Gy at a dose rate of 0.39 Gy/min or 8.25 Gy/min as described in the section above. Cytochalasin B (Sigma-Aldrich, Sweden) was added at a final concentration of 5.6 µg/ml immediately after irradiation and cells were harvested 48 h later. To this end, cells were trypsinised, transferred to centrifuge tubes, spun down and resuspended in warm (37 °C) 0.14 M KCl (Sigma-Aldrich). After a 5 min incubation time at room temperature, the cells were centrifuged and fixed in fixative I (methanol: 0.9% NaCl: acetic acid (Sigma-Aldrich); 12:13:3) and subsequently in fixative II (methanol: acetic acid; 4:1). Following 2–3 washes with fixative II cells were dropped onto clean, dry microscopic slides (Menzel-Glaser, Germany), dried and stained with 5% Giemsa (Merck, Germany) for 10 min. 3 independent experiments were performed. 500 binucleated cells per dose and harvest time point were scored for micronuclei on blinded slides by a single scorer.

### Statistical analysis and curve fitting

95% confidence intervals (CI) were calculated based on standard deviation values from three independent repeat experiments using the *confidence.norm* function of MS Excel (version 2013). Differences between treatments are regarded as significant when the 95% CI of the difference between two mean values did not contain 0 (Gardner and Altman 1986). In accordance with Altman and Krzywinski (2017), who caution against “*P* value hacking”, significant data points on the graphs are not marked.

Dose–response relationships for the levels of mRNA, MN and clonogenic cells survival were fitted using the linear quadratic function *c* + *aD* + *bD*^2^, where *D* = the radiation dose in Gy. The kinetics of foci induction and decay were fitted to the equation *f* = (*a* + *bx*)/(1 + *cx* + d*x*^2^), where *f* is the focus frequency and *x* is the time in min. Fitting was performed using the Marquardt–Levenberg least squares algorithm which is incorporated in the graphic software SigmaPlot 14.0 (Systat Software Inc, USA).

Replication index was calculated according to the formula (1*N*1 + 2*Nx*2 + 3*Nx*3 + 4*Nx*4 + 5*Nx*5)/*n*, where *N* = number of nuclei in a cell and *n* = number of scored cells.

## Results

### Gene expression in leukocytes

**Fig. 1:**
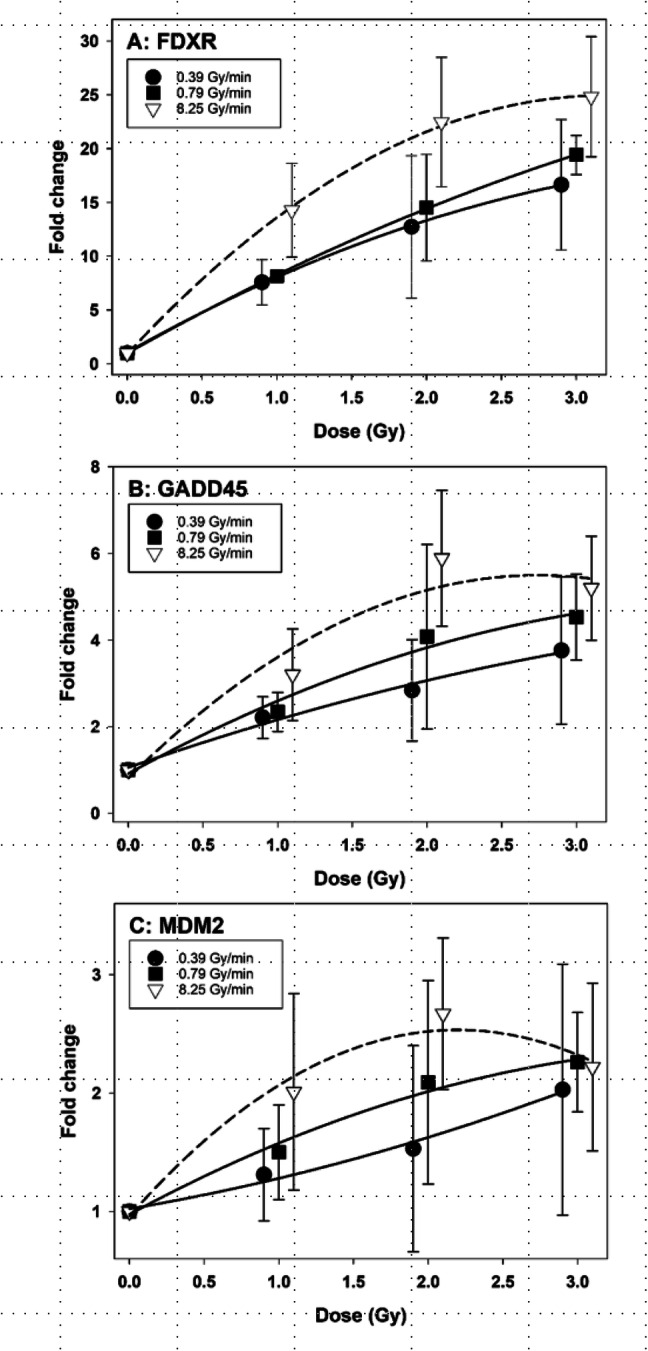
Dose–response curves for relative mRNA levels of genes **a** FDXR, **b** GADD45 and **c** MDM2 in human peripheral blood lymphocytes exposed to gamma radiation at 0.39, 0.79 and 8.25 Gy/min. Data points are nudged to avoid overlap. Error bars: 95% confidence intervals from three independent experiments with lymphocytes of one donor

Whole blood samples were exposed to 0, 1, 2 and 3 Gy of gamma radiation delivered at 0.39, 0.79 and 8.25 Gy/min and mRNA levels of FDXR, GADD45a and MDM2 were analysed by qPCR 24 h later. The results are shown in Fig. 1a–c, separately for each gene. For all three genes, the mRNA levels increased with the dose. The highest fold change was observed for FDXR, followed by GADD45a and MDM2. For every gene, the mRNA levels were directly related to the dose rate. The strongest dose rate effect was observed for 8.25 Gy/min. The single dose rate points were seldom significantly different from each other due to large inter-experimental scatter, but the difference was consistent over the studied dose range. An interesting observation was that the dose–response relationships for radiation delivered at 8.25 Gy/min showed a strong curvature. This was not the case for 0.39 and 0.79 Gy/min.

### Micronuclei in lymphocytes

**Fig. 2:**
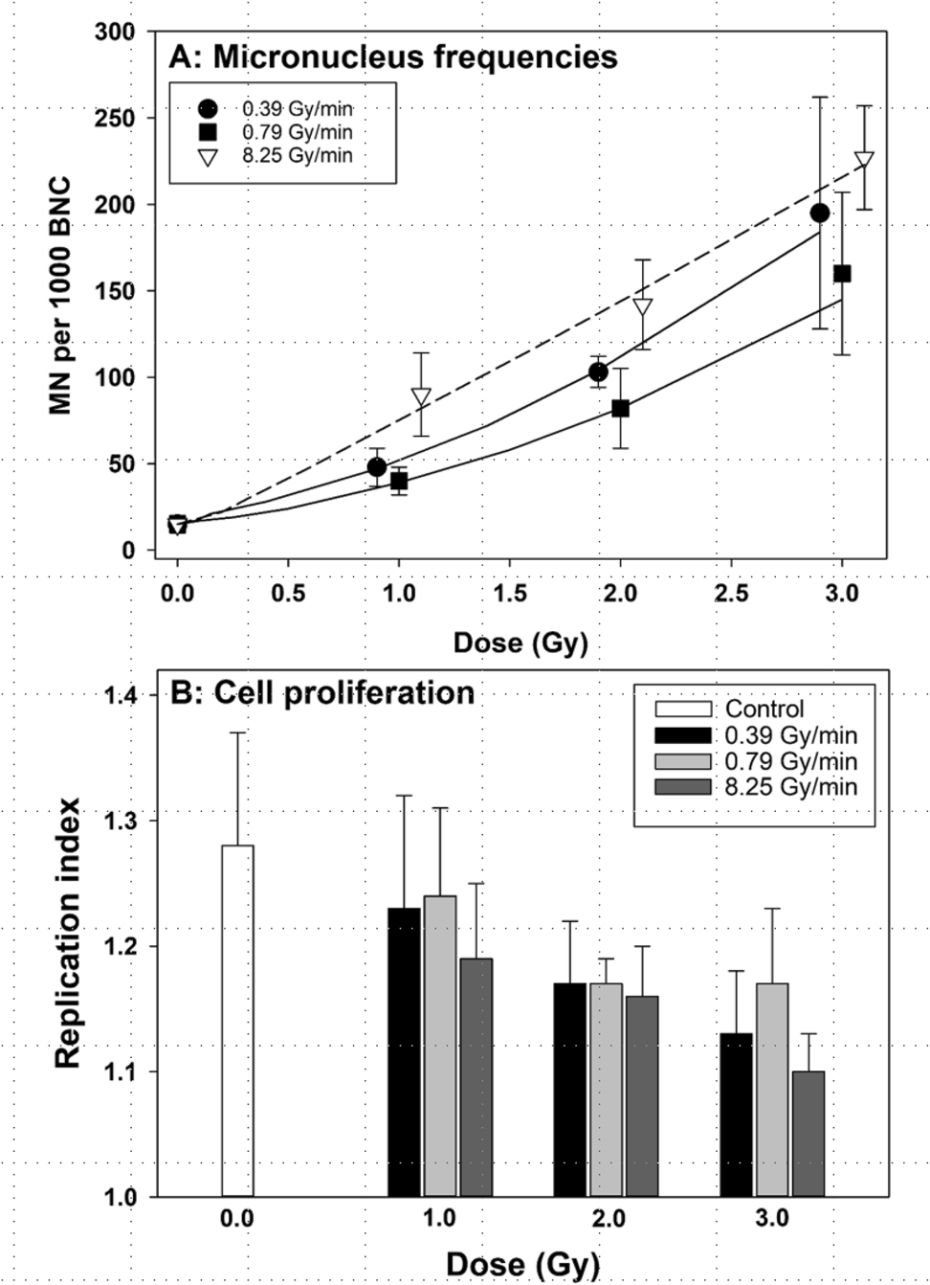
Results of micronucleus analyses in human peripheral blood lymphocytes. **a** Frequency of micronuclei, **b** replication indices. Data points in **a** are nudged to avoid overlap. Error bars: 95% confidence intervals from three independent experiments with lymphocytes of one donor

To compare the impact of dose rate on gene expression with that on cytogenetic damage, whole blood samples were exposed to 0, 1, 2 and 3 Gy of gamma radiation delivered at 0.39, 0.79 and 8.25 Gy/min, and micronuclei and cell proliferation were analysed in cells harvested 72 h later. The results are shown in Fig. 2. Similarly as for gene expression, the highest level of MN was observed in cells exposed at 8.25 Gy/min (Fig. 2a). A trend towards an inversed dose rate effect was seen for dose rates 0.39 and 0.79 Gy/min, although none of the dose rate points differed significantly (not shown). In contrast to the gene expression result, the dose–response curve for 8.25 Gy/min was nearly linear, while the curves for 0.39 and 0.79 Gy/min showed distinct curvatures. Replication indices (RI) are shown in Fig. 2b. Overall, RI values declined with the dose. Consistently, lower values were observed in cells exposed to radiation at 8.25 Gy/min, corresponding with the highest level of MN frequency.

The distributions of MN were analysed to verify whether differences between the MN frequency are representative for the whole cell population. Results are shown in Table 1. Similar values of dispersion indices were observed among all dose and dose rate points, suggesting that this is the case.

**Table 1:** Mean dispersion indices of MN and standard deviations from three independent experiments with lymphocytes of one donor

Dose rate (Gy/min)	Dose (Gy)	DI	SD
0.39	1	1.21	0.09
0.39	2	1.27	0.11
0.39	3	1.40	0.09
0.79	1	1.16	0.05
0.79	2	1.29	0.21
0.79	3	1.24	0.09
8.25	1	1.18	0.13
8.25	2	1.29	0.04
8.25	3	1.40	0.03

### 53BP1 foci in U2OS cells

**Fig. 3:**
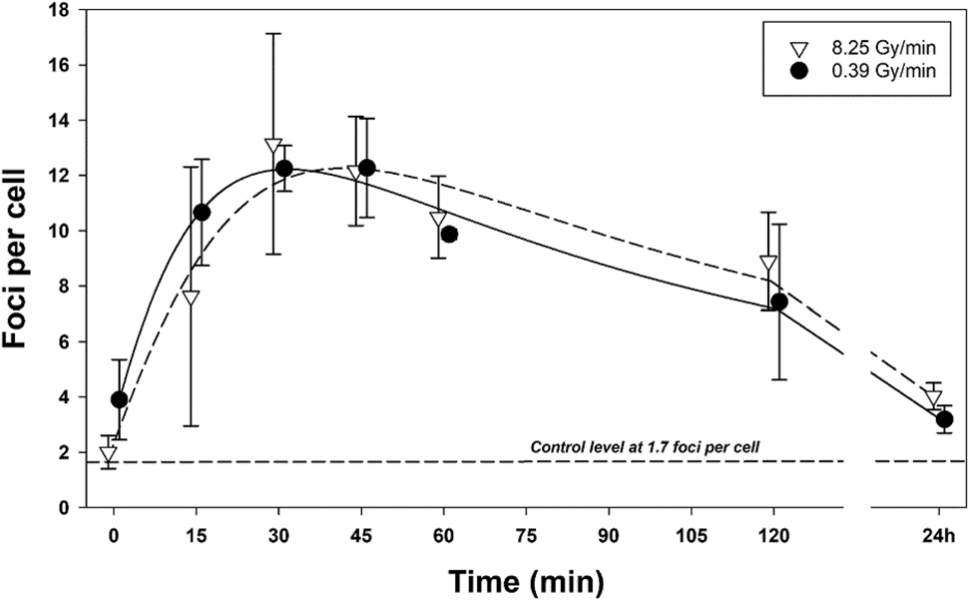
Kinetics of 53BP1 foci formation and decay in U2OS-53BP1 cells exposed to gamma radiation at 0.39 and 8.25 Gy/min. Data points are nudged to avoid overlap. Error bars: 95% confidence intervals from three independent experiments

To validate the results achieved with human lymphocytes, experiments were carried out with U2OS-53BP1 cells that were exposed to 3 Gy of gamma radiation delivered at 0.39 and 8.25 Gy/min, harvested between 0 min and 24 h later and analysed for the frequency and size of 53BP1 foci. The results are shown in Fig. 3.

Radiation delivered at both dose rates induced the maximal frequency of foci around 30 min post-exposure. Thereafter, the frequency of foci decayed, coming close, but not reaching the control level after 24 h. The focus frequencies between 0.39 and 8.25 Gy/min at 0 min and 24 h differ significantly. The fraction of large foci was between 0.1 and 0.34 and did not differ consistently between cells irradiated at the two dose rates (Table 2). For this reason, the kinetics of focus formation and decay is shown in Fig. 3 for all foci.

**Table 2:** Fraction of large foci in U2OS cells cells exposed to gamma radiation at 0.39 and 8.25 Gy/min and standard deviations (SD) from three independent experiments with lymphocytes of one donor

Time p.r. (min)	8.25 Gy/min		0.39 Gy/min	
	Mean	SD	Mean	SD
0	0.26	0.05	0.10	0.03
15	0.16	0.03	0.16	0.01
30	0.17	0.04	0.20	0.04
45	0.19	0.05	0.20	0.05
60	0.20	0.04	0.26	0.05
120	0.19	0.04	0.26	0.08
24 h	0.34	0.12	0.25	0.10

Controls mean: 0.29, SD: 0.07

### Clonogenic cell survival and micronucleus frequencies in U2OS cells

**Fig. 4:**
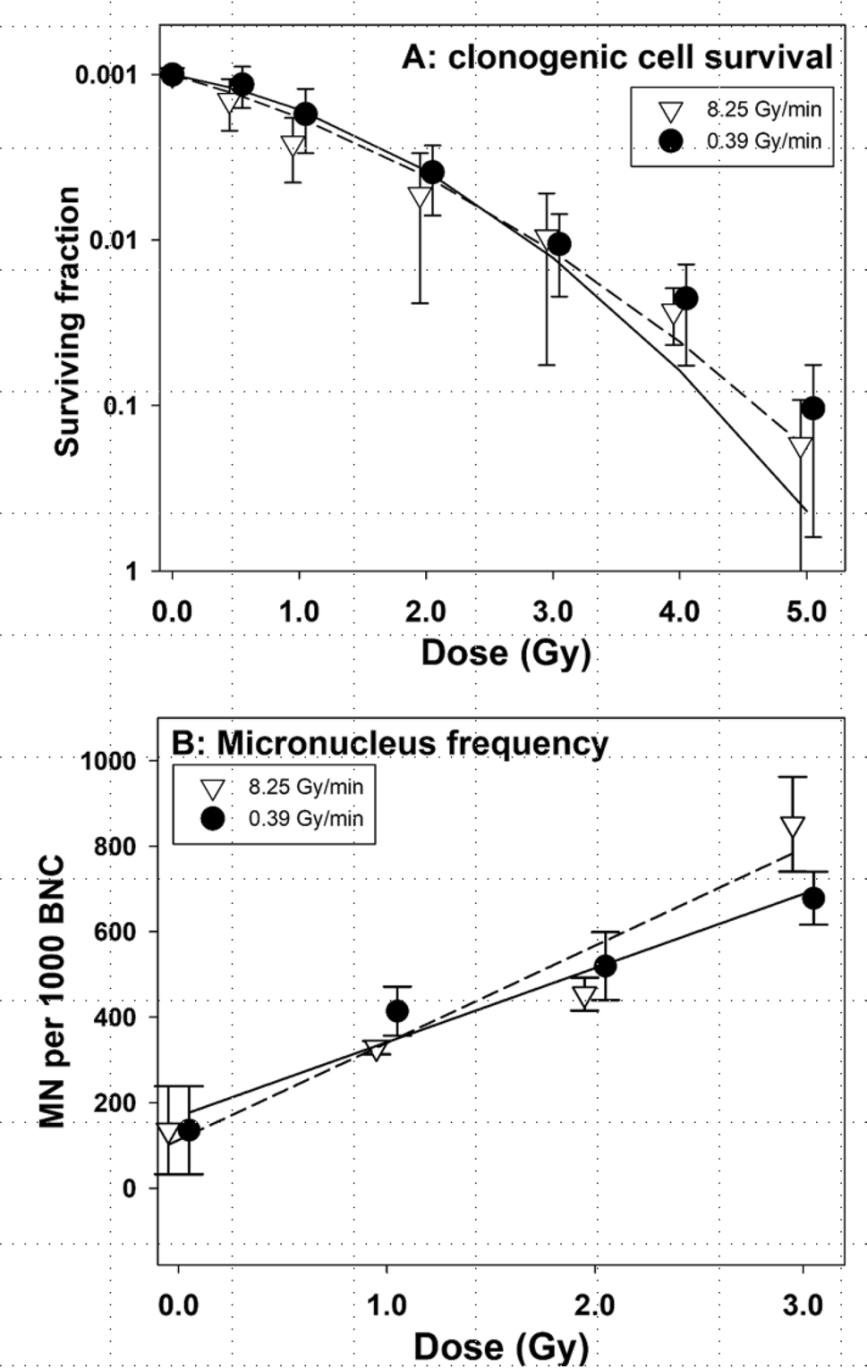
Clonogenic cells survival (**a**) and micronucleus frequencies (**b**) in U2OS cells exposed to gamma radiation at 0.39 and 8.25 Gy/min. Data points are nudged to avoid overlap. Error bars: 95% confidence intervals from three independent experiments

Results of clonogenic cells survival test in U2OS-53BP1 cells are shown in Fig. 4a. No consistent difference was observed between cells exposed to radiation at the two dose rates.

Frequencies of MN were analysed in cells exposed to increasing doses of radiation at the two dose rates and harvested at 48 h post-exposure. The results are shown in Fig. 4b. Similarly as for clonogenic cell survival, no difference in the MN yields was observed between cells exposed to radiation at the two dose rates.

## Discussion

The aim of this investigation was to compare the biological effectiveness of gamma radiation delivered at VHDR (8.25 Gy/min) with that of HDR (0.38 Gy/min or 0.79 Gy/min). Experiments were carried out with human peripheral blood lymphocytes and the human osteosarcoma cell line U2OS. Endpoints related to DNA damage response were analysed. The results obtained with human peripheral blood lymphocytes clearly show that gamma radiation delivered at VHDR is more effective in inducing DNA damage than when delivered at HDR. The results obtained with U2OS cells are less clear.

Experiments were carried out with two cell systems: human peripheral blood mononuclear cells (PBMC) and U2OS-53BP1 cells. The reason for choosing PBMC was that they are extensively used for the purpose of retrospective biological dosimetry (Wojcik et al. 2017) and for testing biomarkers of individual radiosensitivity (Rajaraman et al. 2018). The reason for choosing U2OS-53BP1 cells was that they constitutively express the 53BP1 protein, allowing analysis of ionising radiation-induced repair foci (IRIF) (Sollazzo et al. 2017) without antibody labelling. Moreover, U2OS cells readily form colonies allowing analysis of clonogenic cell survival (Franken et al. 2006). We chose to analyse PBMC from one donor for each assay to exclude the problem of inter-donor variability (Cheng et al., 2019). The observation that both assays show a higher effectiveness of VHDR as compared to HDR in inducing DNA damage suggests that the effect is not donor specific. However, a higher number of donors per assay need to be analysed to reveal the true level of inter-donor variability. The lack of a clear VHDR effect in U2OS cells suggests that the response may be cell-type specific, but the present study was not designed to answer this question. Rather, it can be regarded as a pilot study that highlights the necessity to analyse the problem of VHDR in greater detail.

Two endpoints were analysed in PBMC: expression levels of three known radiation-responsive genes (Li et al. 2017) and the frequency of micronuclei in binucleated cells (Fenech 2000; Müller and Streffer 1994). Results obtained with both endpoints clearly point towards an increased effectiveness of VHDR in inducing cellular damage. For gene expression, it is most pronounced for the FDXR gene, which is known to be a particularly sensitive biomarker of radiation exposure (O'Brien et al. 2018). Overall, the VHDR/HDR ratio is highest in the dose range of 1–2 Gy (with a value of about 2) and declines thereafter. The reason for the decline is differences in the shapes of the dose–response curves: for gene expression, the VHDR dose–response relationship is linear quadratic (saturating with increasing dose) and HDR is (mostly) linear. For MN, the VHDR dose–response relationship is linear while for HDR—linear quadratic. The dose–response curves are constructed based on three dose points only, so need to be treated with caution, but the results are worth stressing because they are consistent across the assays. Moreover, this pattern is highlighted because the dose–response curves for VHDR resemble those observed after exposure of PBMC to radiation of high linear energy transfer (LET) that is known to induce a high level of difficult to repair, complex DNA damage (Goodhead 2006). For MN in PBMC, upward linear-quadratic dose–response relationships are common for low LET radiation like photons (Depuydt et al. 2017), while linear or saturating dose–response relationships are observed for alpha particles (Johannes et al. 2010; Mill et al. 1996). For gene expression, the opposite is observed: the dose–response is downward linear quadratic for alpha particles and linear for photons (Cheng et al. 2019). Thus, it can be postulated that radiation at VHDR induces more difficult to repair, complex damage than at HDR, resembling the situation following high and low LET radiation exposure, respectively. This conclusion fits well with recently reported effects of ultra-high dose rate (UHDR) on melanoma cells (Buontempo et al. 2018). Also, the presented results support the recent multiparametric study, demonstrating a higher biological effectiveness of 4 MV photons delivered at a dose rate of 2.5 Gy min^−1^ as compared to 0.63 Gy min^−1^ (Ben Kacem et al. 2020).

The conclusion based on results obtained in PBMC is partly substantiated by results obtained in U2OS cells. The kinetics of IRIF formation and decay was analysed up to 24 h post-irradiation. IRIF induced by VHDR formed with a delay and persisted longer than those induced by HDR. The effect is not strong, but consistent. The result is in line with the observation of delayed formation and decay of IRIF in U2OS cells exposed to alpha particles as compared to photons (Sollazzo et al. 2017) and corroborates the conclusions from results observed in PBMC.

Clonogenic cell survival and the frequency of MN were analysed in U2OS cells and here no difference was detected between VHDR and HDR. This may not be surprising in view of the weak effect seen at the level of 53BP1 foci. Why the VHDR effect was weaker in U2OS cells as compared to PBMC is not clear, but may be related to the fact that the former are transformed while the latter are not. Obviously, the present results must be validated in experiments using different cell lines and more peripheral blood donors.

In contrast to PBMC, the MN dose–response curves for both VHDR and HDR were linear in U2OS cells which are related to the fact that the cells were irradiated during asynchronous growth, while PBMC were irradiated in the G_0_ phase of the cell cycle. The dose–response relationships for cytogenetic damage in cells irradiated during exponential growth are usually linear because of cell cycle perturbations and varying radiation sensitivity of various phases of the cell cycle (Galecki et al. 2019; Kaufman et al. 1974; Savage and Papworth 1973; Shibamoto et al. 1994).

Before discussing the significance of the results for radiological protection in the context of other published studies, it is necessary to recall that radiation effects are categorised in two groups: deterministic effects, also termed tissue effects, that are caused by cell death and stochastic effects that are caused by changes in the genome which may lead to the development of cancer in cells whose ability to proliferate was not compromised (ICRP 103 2007). Numerous results have demonstrated that reducing the dose rate below the HDR range exerts a sparing effect on cell killing due to improved repair of sub-lethal damage (Badie et al. 1996; Bedford et al. 1978). For the same reason, the frequency of unstable chromosomal aberrations and micronuclei is reduced per unit dose when the dose rate is reduced below HDR (Bauchinger et al. 1979; Manesh et al. 2014; Tanaka et al. 2009; Vral et al. 1992).

With respect to stochastic effects, the sparing effect of reducing the dose rate below the HDR range is far less clear. This problem is now in the focus of interest, because of discussions regarding the validity of the DDREF (Ruhm et al. 2016, 2018). Because epidemiological studies on cohorts exposed to low dose rate radiation are not able to provide a clear answer (Hoel 2018), studies have been carried out with animals and in vitro cell systems. Initial studies on mice, the famous mega-mouse experiments carried out in the USA after World War II, revealed a clear sparing effect on mutation frequencies when the dose rate was reduced below HDR (Russell et al. 1958). Later, animal studies on cancer and non-cancer stochastic effects confirmed the existence of DDREF (reviewed in Little 2018). Results of cell experiments which focused on mutations and stable-type chromosomal aberrations that can be regarded as molecular events related to cancer (Beroukhim et al. 2007) are contradictory, with some showing no or little sparing effect of LDR (Evans et al. 1990; Manesh et al. 2014) and other demonstrating clear effects (Kiefer et al. 2002) (reviewed in Manesh et al. 2014).

In all the above studies that were triggered by the desire to validate the LSS-derived risk factors in a chronic exposure scenario, the biological effectiveness of LDR radiation was compared to HDR radiation that was not higher than ca 1.0 Gy/min. Exemplary HDR values were 0.3 Gy/min (Grosovsky and Little 1985), 0.3 Gy/min (Ueno et al. 1982), 0.4 Gy/min (Manesh et al. 2014), 0.5 Gy/min (Furuno-Fukushi et al. 1988), 0.5 Gy/min (Lorenz et al. 1994), 0.5 Gy/min (Nakamura and Okada 1981), 0.70–0.79 Gy/min (Russell et al. 1958), and 0.83 Gy/min (Evans et al. 1990). These values are much lower than the tens of Gy/min following the atomic bomb detonations in Hiroshima and Nagasaki (Ruhm et al. 2018). The results presented in this report suggest that the biological effectiveness of gamma radiation increases when the dose rate reaches ca 8 Gy/min. Hence, the DREF value may be underestimated when a dose rate of 1 Gy/min is used in cell or animal experiments to compare the biological effectiveness of HDR. It is possible that different DREF values should be applied when transferring risk estimates from VHDR and HDR exposures to LDR scenarios.

Finally, the VHDR effect described in this study is not only relevant for estimating the DREF value. The analysis of chromosomal aberrations in PBMC is the gold standard of retrospective biological dosimetry (Ainsbury et al. 2011). The absorbed dose is estimated by comparing the yield of chromosomal aberrations of a radiation accident victim with a calibration curve which is generated by ex vivo irradiation of PBMC drawn from a healthy donor with known doses of radiation. The recommended dose rate for generating the calibration curve is 1 Gy/min (IAEA 2011). In cases when the dose rate of the accidental exposure was in the VHDR range, the assessed dose may be overestimated.
